# Models for predicting short- to long-term mortality in older adults with hip fractures in clinical practice: a systematic review and meta-analysis

**DOI:** 10.1186/s12877-026-07814-y

**Published:** 2026-06-23

**Authors:** Kazuhiro Miyata, Shuntaro Tamura, Sota Kobayashi, Hiroyuki Saito, Yoichi Kaizu

**Affiliations:** 1 Department of Physical Therapy, Ibaraki Prefectural University of Health Science, 4669-2, Ami-Machi, Inashiki-gun, 300-0394 Ibaraki Japan; 2 Department of Physical Therapy, Ota College of Medical Technology, Gunma, Japan; 3 Department of Physical Therapy, Niigata University of Health and Welfare, Niigata, Japan; 4 Department of Rehabilitation, Geriatrics Research Institute and Hospital, Gunma, Japan; 5 Department of Rehabilitation Center, Hidaka Rehabilitation Hospital, Gunma, Japan

**Keywords:** Death, Prediction model, Prognostic model, Fracture

## Abstract

**Background:**

Despite published summaries and appraisals of prognostic models for mortality after hip fracture, it remains unclear which models should be used with the evidence obtained to date. We systematically reviewed validated prognostic models for predicting mortality in older adults with hip fractures.

**Methods:**

A search of Medline, CINAHL, The Cochrane Library, CiNii, and Ichushi databases on April 2024. Model development with internal or external validation studies and external model validation studies of previously reported models were selected. Models predicting mortality outcomes at short- to long-term follow-up time points were included. We used the PROBAST instrument to assess the risk of bias and synthesized the studies’ data to perform a meta-analysis. A structured five-step method was applied to determine which prognostic models were clinically valuable.

**Results:**

After screening 1,092 publications, we identified 24 studies (21 prognostic models) for this review. The model discrimination, measured by the area under the curve or C-statistic, ranged from 0.68 to 0.89 for the development models and 0.66–0.84 for the validation models. With the exception of the four validation models, all development and validation models were deemed to be at high risk of bias. Common concerns were participant and analysis domains. Our meta-analysis of the Nottingham Hip Fracture Score in 30-day mortality revealed a pooled C-statistic at 0.68 (95%CI: 0.66–0.71). A narrative synthesis identified the Physiological and Operative Severity Score for the enUmeration of Mortality and morbidity and a mortality prediction score as predictors of in-hospital mortality and the Nottingham Hip Fracture Score as a predictor of 1-year mortality.

**Conclusions:**

These results suggest that, based on the current evidence, recommended prognostic models for predicting mortality at each time point. However, given the high risk of bias in most studies, these models should be used with caution and only as adjunctive tools. Future studies should prioritize external validation and reducing the risk of bias.

**Trial registration:**

PROSPERO CRD42023462537.

**Supplementary Information:**

The online version contains supplementary material available at https://doi.org/10.1186/s12877-026-07814-y.

## Background

Hip fracture is one of the most serious types of osteoporotic fracture worldwide, with an approx. 20% first-year mortality rate [1]. Coupled with the increasing number of hip fractures associated with demographic aging, the prediction of core outcome sets after fractures [2] including mortality, disability, and quality of life is of public health significance. Prognostic models are valuable tools for health professionals to facilitate shared decision-making regarding surgery, treatment, and/or rehabilitation after hip fracture, and there has been increasing interest in prognostic models that can be used for older adults with hip fractures.

To date, many prognostic models have been developed to predict patients’ recovery after hip fractures, focusing on in-hospital and 30-day mortality [3–5]. Typical prognostic models include the Nottingham Hip Fracture Score (NHFS) [6] and the Physiological and Operative Severity Score for the enUmeration of Mortality and morbidity (POSSUM) score [7], which clinicians can obtain from patients before or during surgery. To be clinically useful, prognostic models should be rigorously developed and validated to ensure their accuracy and generalizability [8–10]. Unfortunately, many of the existing prognostic models are poorly developed and optimistically reported (~ 30% do not provide the final model) [11]. The implementation of new prognostic models in clinical practice requires external validation and an impact assessment after the models’ development [8–10]. Without validation, the likelihood of a prognostic model being used in clinical practice is limited. Nevertheless, according to previous reviews, only 4.6%–36% of models with external validation and 0%–0.2% of models with an impact assessment have been implemented [12, 13].

Although summaries and appraisals of prognostic models for hip fracture have been published, it remains unclear which models should be used with the evidence obtained to date. This systematic review aims to implement at each time point an externally validated prognostic model for predicting mortality in older adults with hip fractures. Furthermore, the identified prognostic model should be externally validated in the future.

## Methods

We conducted a systematic review in accord with the Transparent Reporting Of multivariable prediction models for Individual Prognosis Or Diagnosis checklist for Systematic Reviews and Meta-Analyses (TRIPOD-SRMA) [14] and the Preferred Reporting Items for Systematic Review and Meta-Analyses (PRISMA) 2020 statement [15]. The present systematic review was registered with the PROSPERO database (registration no. CRD42023462537). An ethics statement was not required because this study was based exclusively on published research.

### Study eligibility criteria

We included studies that met the following criteria.

- Type: Three types of studies were included: prognostic model development studies with internal validation, prognostic model development studies with external validation in independent data reported in the same study, and external model validation studies of previously reported models. Studies describing only the development of a prognostic model were excluded.
- Design: Prospective or retrospective cohort studies
- Participants: Inpatients after hip fracture surgery who were ≥ 60 years old
- Outcome: Mortality
- Period of prediction: No restrictions

The exclusion criteria were as follows: studies (*i*) that did not concern a multifactorial prognostic model including two or more predictors (e.g., risk factor studies and randomized control trials were excluded), (*ii*) the subjects were < 60 years old, (*iii*) inpatients were not included, (*iv*) participants other than those who underwent hip fracture surgery were included, (*v*) individuals with Parkinson’s disease or stroke were included, and (*vi*) studies that were of a different study type (e.g., conference presentation, review article, or letter to the editor) even if the model was built.

### Information sources

On April 16, 2024, we searched five electronic bibliographic databases: Medline (via PubMed), CINAHL (Ebsco platform), The Cochrane Library, CiNii (a Japanese database), and Ichushi (a Japanese database). Publications from the 20-year period immediately prior to the search date were included.

### Search strategy

We developed a search strategy using a combination of free text and Medical Subject Heading terms for hip fracture, mortality, and prognostic models (Supplemental Table 1).

### Study selection process

All retrieved records were imported into EndNote X9 for management, and duplicates were removed. Titles and abstracts were screened by KM and another author independently (YK, HS, SK, or ST). Full texts were retrieved for all potentially eligible studies and reviewed by KM and another author (YK, SK, or ST) independently, to determine the texts’ eligibility. Reasons for the exclusion of full texts were recorded. Disagreements were resolved through discussion.

### Data collection process

We used the Checklist for Critical Appraisal and Data Extraction for Systematic Reviews of Prediction Modelling Studies (CHARMS) [16] to guide the data extraction from published papers and supplemental materials. Two reviewers (KM and YK) independently extracted the data from the retained studies. In cases of disagreement, a third reviewer (ST) was brought in to reach a consensus. Study authors were contacted by email for additional information that may not have been included in the original articles.

### Data items

To describe the study characteristics, we extracted the information on each study’s first author, publication year, study location, study design, source of data, number of participants, outcome(s) definition, and measurement(s) of the outcome. To describe the performance of the model, we extracted information on any reported measure related to discrimination (e.g., the area under the receiver operating characteristic curve [AUC], the C-statistic), calibration (e.g., the calibration plot), diagnostic accuracy, and/or clinical utility [17]. ‘Discrimination’ measures how well the model distinguishes between individuals who died and those who survived. ‘Calibration’ reflects the agreement between observed events and expected events (i.e., are the predictions accurate) and is a critical performance feature for clinical use. A prognostic model may have good discrimination, meaning individuals who died have higher predicted risk scores than those who survived, yet produce poorly calibrated (inaccurate) predictions.

### Risk of bias and applicability assessment

We assessed each study’s risk of bias by using the Prediction model Risk of Bias Assessment Tool (PROBAST) [18] as advised by the Cochrane Prognosis Methods Group for prognostic reviews. The risk of bias was evaluated according to four domains (participants, predictors, outcomes, and analysis), and applicability was evaluated according to three domains (participants, predictors, and outcomes). The risk of bias was categorized as high, low, or unclear based on 20 signaling questions.

‘Applicability’ refers to the degree to which the three domains of the study under review are consistent with a pre-established review question predetermined by the reviewer. If the agreement with the review question is low, the evaluation is considered a “high applicability concern.” Usually, one review question is established per systematic review. Our review question concerned: (*i*) the population: older adults (≥ 60 years old) after hip fracture surgery; (*ii*) the outcomes: mortality (timing of measurement not specified); and (*iii*) the domains of the predictors: demographic, medical history/comorbidities, fracture information, surgical information, biochemical markers, cognitive function, physical function, and psychiatric information (timing of measurement: immediately after surgery).

The results of our analyses of the risk of bias and applicability were appraised independently by KM and another author (YK, HS, SK, or ST). Independence between the reviewers was ensured by using a separate Excel sheet for the evaluation that blocked access to the other reviewer’s responses. Disagreements were resolved through discussion with a third reviewer, and consensus was reached on all studies.

### Synthesis methods

The data were summarized with the use of descriptive statistics and visualizations. We conducted a meta-analysis if the same prognostic model had more than five external validations at one time point [19]. Based on previous recommendations [20], we pooled the logit C-statistic by conducting a random-effects meta-analysis. We calculated 95% confidence intervals (CIs) to quantify uncertainty and the presence of between-study heterogeneity. The 95%CI indicates the precision of the summary performance estimate. The primary analysis included only studies with complete data, and sensitivity analyses were performed by additionally including studies with supplemented data. All analyses were conducted in R ver. 4.4.1 using the ‘metafor’ packages.

For time points at which a prognostic model was externally validated but could not be meta-analyzed, we performed a narrative synthesis focusing on the description of model characteristics. To guide clinicians in their decision regarding whether to use these prognostic models according to their specific practice contexts, we adopted the structured five-step method proposed by Naye et al. [21] for determining which prognostic models are clinically valuable: for a prognostic model to show clinical value, the following five criteria must be fulfilled.

1. Low concerns about applicability.
2. A complete report of performance measures is available.
3. Calibration performance measures must be acceptable or good (calibration slope = 1, calibration intercept = 0, and/or a calibration plot considered good or acceptable).
4. Discrimination performance measures must be between 0.61 and 0.75 to be possibly helpful and > 0.75 to be clearly helpful for clinicians.
5. The risks of bias must have minimal impact on the model. For example, a non-appropriated data source is more detrimental than predictors that are part of the outcome definition.

## Results

### Study selection

**Fig. 1:**
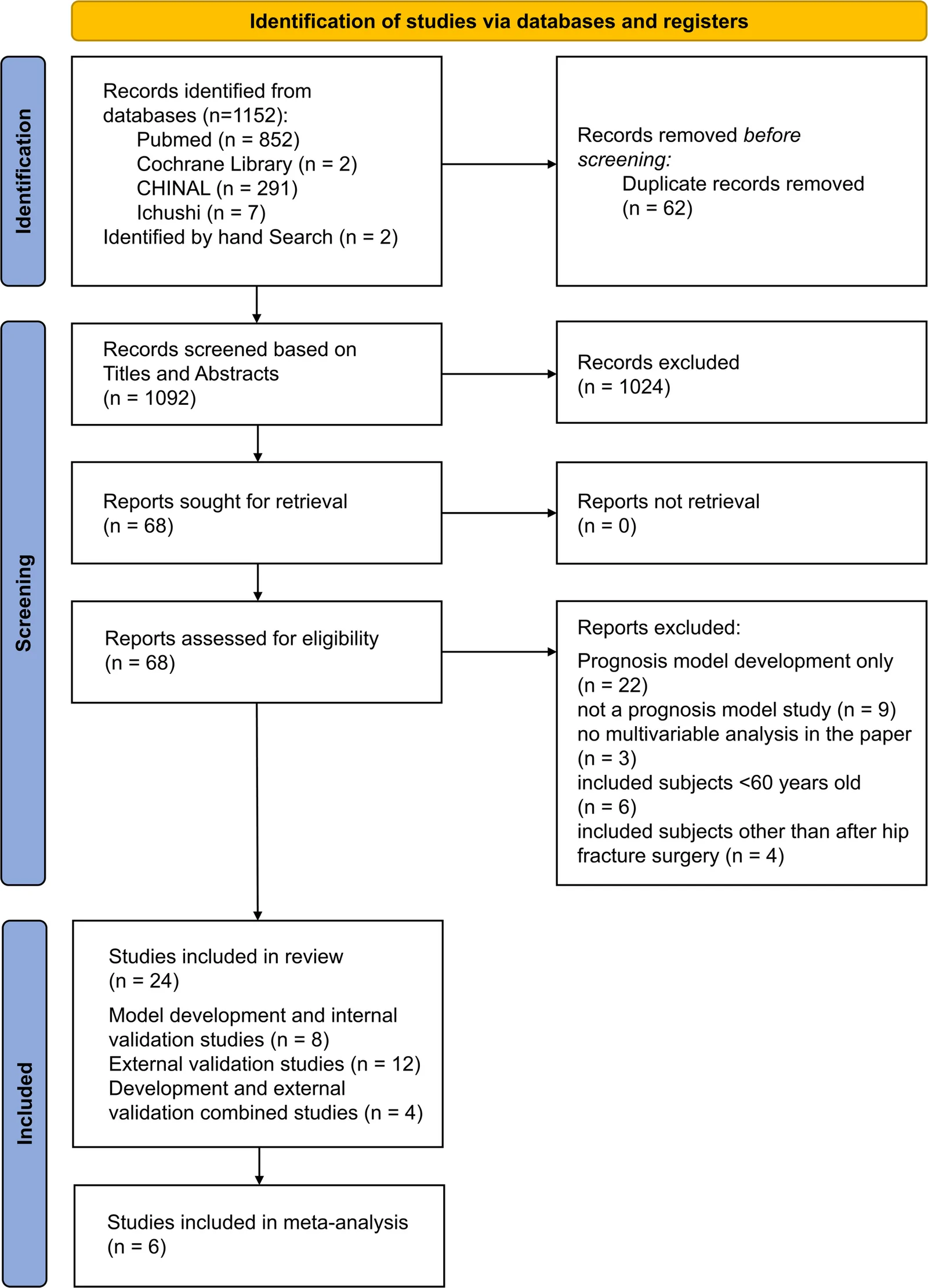
Flow of studies for this systematic review

After the removal of duplicates, we identified 1,092 studies for title and abstract screening. Of these, 68 studies were deemed potentially relevant; 24 studies [22–45] ultimately met the eligible criteria (including 21 prognostic models in total) (Fig. 1).

### Characteristics of included studies and models

Table 1 describes the main characteristics of the included studies. Of the 24 included studies, nine studies concerned the development and internal validation (eight models [23, 24, 27, 29, 34, 36, 43, 45]), while 12 studies were external-validation studies only (nine models [22, 25, 26, 31–33, 35, 37, 38, 41, 42, 44]), and the remaining four studies combined development and external validation in one publication (four models [28, 30, 39, 40]). Temporal validation (*n* = 3, 18.8%) and geographical validation (*n* = 13, 81.2%) were the two main methods for external validation.

**Table 1:** Individual study characteristics

Study (ref.)	Prediction(s) model(s)	Country	Design	Source of data	*n* at baseline (*n*, % at follow-up)
Development					
Cary Jr 2021 [23]	Cary Jr. clinical prediction model	USA	retrospective cohort	Medicare registry	17,140 (17,140, 100%)
Cenzer 2016 [24]	Cenzer clinical prediction model	USA	retrospective cohort	Health and Retirement Study database	1,124 (857, 76%)
Fu 2021 [27]	Fu clinical prediction model	China	retrospective cohort	One hospital	874 (702, 80%)
Hjelholt 2022 [29]	Hjelholt clinical prediction model	Denmark	retrospective cohort	Danish Multidisciplinary Hip Fracture Registry	42,178 (28,791, 68%)
Nijmeijer 2023 [34]	AHFS^90^	Netherlands	retrospective cohort	Dutch Hip Fracture Audit database	922 (922, 100%)
Oosterhoff 2022 [36]	SORG femoral neck fracture mortality algorithm	Netherlands	retrospective cohort	Research Patient Data Registry	2,478 (2,478, 100%)
Yoo 2022 [43]	Yoo clinical prediction model	Korea	retrospective cohort	Korea National Healthcare Insurance Service database	196,842 (196,842, 100%)
van de Ree 2019 [45]	BHFS	Netherlands	retrospective cohort	Two hospitals	925 (916, 99%)
Development and validation					
Goubar 2023 [28]	Stratify-Hip algorithm	UK	retrospective cohort	National Hip Fracture Database	In-hospital Development: 170,411 (141,158, 83%) Validation: 90,102 (84,096, 93%) 30-day Development: 170,411 (149,258, 88%) Validation: 90,102 (87,414, 97%)
Jiang 2005 [30]	Jiang Risk score	Canada	retrospective cohort	Two hospitals	Development: 2,187 (2,187, 100%) Validation (1,794, 100%)
Sanz-Reig 2024 [39]	RNFC model	Spain	retrospective cohort	Spanish RNFC database	Development: 16,427 (16,427, 100%) Validation: 13,448 (13,448, 100%)
Schoeneberg 2023 [40]	GeRi-Score	Germany Switzerland Austria	retrospective cohort	ATR-DGU	Development: 20,602 (19,054, 92%) Validation: 21,011 (19,516, 93%)
Validaiton					
Arjan 2023 [22]	OPERA score NHFS	UK	retrospective cohort	Two hospitals	2,142 (2,142, 100%)
Doherty 2021 [25]	NHFS	UK	retrospective cohort	National Hip Fracture Database	3,208 (3,092, 96%)
Etscheidt 2021 [26]	MPS	USA	prospective cohort	Five hospitals	2,296 (1,376, 60%)
Jonsson 2018 [31]	POSSUM P-POSSUM NHFS	Sweden	prospective cohort	One hospital	1,556 (997, 64%)
Karres 2022 [32]	NHFS HEMA	Netherlands	prospective cohort	Two hospitals	244 (243, 99%)
Marufu 2016 [33]	NHFS	UK	retrospective cohort	ASAP data	10,908 (9,017, 83%)
Olsen 2021 [35]	NHFS	Sweden	retrospective cohort	Two hospitals	1,864 (1,864, 100%)
Oosterhoff 2023 [37]	SORG femoral neck fracture mortality algorithm	Israel	retrospective cohort	One hospital	2,033 (2,033, 100%)
Rushton 2015 [38]	NHFS	UK	retrospective cohort	Two hospitals	1,118 (1079, 97%)
Schuijt 2022 [41]	U-HIP	USA	retrospective cohort	NSQIP	25,502 (25,502, 100%)
Tilkeridis 2018 [42]	NHFS Modified NHFS	Greece	retrospective cohort	One hospital	385 (349, 91%)
van Zeeland 2011 [44]	POSSUM	Netherlands	retrospective cohort	One hospital	272 (271, 99%)

*AHFS*^*90*^ Almelo Hip Fracture Score 90, *ASAP* Anaesthesia Sprint Audit of Practice, *ATR-DGU* Registry for Geriatric Trauma-German Trauma Society, *BHFS* Brabant Hip Fracture Score, *GeRi-Score* Geriatrics at Risk Score, *HEMA* Hip fracture Estimator of Mortality Amsterdam, *MPS* mortality prediction score, *NHFS* Nottingham hip fracture score, *NSQIP* American College of Surgeons National Surgical Quality Improvement Program, *OPERA* Older Persons' Emergency Risk Assessment, *POSSUM* Physiological and Operative Severity Score for the enUmeration of Mortality and Morbidity, *P-POSSUM* Portsmouth-Physiological and Operative Severity Score for the enUmeration of Mortality and Morbidity, *RNFC* National hip fracture registry, *SORG* SORG Orthopaedic Research Group, *U-HIP* Utrecht Hip algorithm

The studies were conducted between 2005 and 2024 in 14 countries: 18 from Europe, five from North America, and three from Asia. Only three of the studies (12.5%) were published before the TRIPOD reporting guidelines (2015), and 33.3% of the studies were published before the PROBAST (2019). Of the 24 studies, 20 (83.3%) were retrospective cohort designs, and four were prospective cohort designs. About half of the studies’ data sources were from one or more institutions (50.0%), and half were from routinely collected data (50.0%). The sample size ranges from 244 to 2,296 individuals for the institutional-based studies and 922 to 196,842 individuals for the database-based studies.

The included studies described 21 different prognostic models to predict the prognosis of death after hip fracture: eight models were in the development stage [23, 24, 27, 29, 34, 36, 43, 45] and 13 were in the validation stage [22, 25, 26, 28, 30–33, 35, 37–42, 44]. Only two prognostic models had multiple external validations: seven for NHFS [25, 31–33, 35, 38, 42] and two for POSSUM [31, 44].

Table 2 summarizes the outcomes and related measurements used in each study. The incidence of the study outcome ranged from 2.3% to 59.4%. The timing of mortality assessment ranged from a minimum of in-hospital mortality to a maximum of 5 years, with most studies using 30 days as the time point. All but two of the studies reported the performance of the model discrimination and calibration. The AUC or C-statistics ranged from 0.68 to 0.89 for the development models and from 0.66 to 0.84 for the validation models. Calibration slopes were reported in 10 studies.

**Table 2:** The prognostic models’ outcome and performance

Study (ref.)	Prediction(s) model(s)	Definition of outcome	Model performance: discrimination	Model performance: calibration
Development				
Cary Jr 2021 [23]	Cary Jr. clinical prediction model	30-day and 1-year mortality	30-day: AUC 0.765, 95%CI: 0.741–0.787 1-year: AUC 0.758, 95%CI: 0.741–0.764	30-day: Slope = 0.990 1-year: Slope = 1.09
Cenzer 2016 [24]	Cenzer clinical prediction model	1-year mortality	C-statistic = 0.73	The ratio of observed and predicted probability showed good calibration
Fu 2021 [27]	Fu clinical prediction model	1-year mortality	C-statistic = 0.789	The plots showed slightly lower calibration with the middle to low risk patients
Hjelholt 2022 [29]	Hjelholt clinical prediction model	1-year mortality	AUC 0.74, 95%CI: 0.73–0.75	Intercept = − 0.01 and slope = 1.01 The plots showed slightly overestimating with the highest risk patients
Nijmeijer 2023 [34]	AHFS^90^	30-day mortality	AUC 0.74, 95%CI: 0.72–0.76	The plots showed overestimating with the moderate- to high-risk patients
Oosterhoff 2022 [36]	Oosterhoff clinical prediction model	90-day and 2-year mortality	90-day: C-statistic = 0.74, 95%CI: 0.67–0.80 2-year: C-statistic = 0.70, 95%CI: 0.63–0.75	90-day: Intercept = − 0.05, 95%CI: −0.37–0.26 and slope = 1.11, 95%CI: 0.73–1.51 2-year: Intercept = − 0.03, 95%CI: −0.27–0.19 and slope = 0.89, 95%CI: 0.62–1.19
Yoo 2022 [43]	Yoo clinical prediction model	In-hospital and 1-year mortality	In-hospital: C-statistic = 0.692, 95%CI: 0.683–0.700 1-year: C-statistic = 0.675, 95%CI: 0.670–0.679	The figure for the ratio of observed and predicted probability showed good calibration
van de Ree 2019 [45]	BHFS	30-day and 1-year mortality	30-day: AUC 0.74, 95%CI: 0.68–0.79 1-year: AUC 0.77, 95%CI: 0.73–0.80	30-day: The plots showed good calibration. 1-year: The plots showed good calibration
Development and validation				
Goubar 2023 [28]	Stratify-Hip Algorithm	In-hospital and 30-day mortality	In-hospital AUC 0.731, 95%CI: 0.726–0.737 (development set), 0.731, 95%CI: 0.727–0.742) (validation set) 30-day AUC 0.711, 95%CI: 0.706–0.716 (development set), 0.712, 95%CI: 0.706–0.727 (validation set)	The plot was good calibration and showed no under or overestimating
Jiang 2005 [30]	Jiang clinical prediction model	In-hospital and 1-year mortality	In-hospital: C-statistic = 0.83 (development set), 0.82 (validation set) 1-year: C-statistic = 0.75 (development set), 0.74 (validation set)	Not reported
Sanz-Reig 2024 [39]	RNFC model	30-day mortality	C-statistic = 0.886 (Development set)	The ratio of observed and predicted probability = 1.09 (validation set)
Schoeneberg 2023 [40]	GeRi-Score	In-hospital mortality	AUC 0.727, 95%CI: 0.711–0.742 (development set), 0.718, 95%CI: 0.703–0.733 (validation set)	The ratio of observed and predicted probability showed a slight underestimation with the moderate and high risk patients
Validation				
Arjan 2023 [21]	OPERA score NHFS	30-day and 1-year mortality	30-day OPERA score: AUC 0.75, 95%CI: 0.73–0.77 NHFS: AUC 0.68, 95%CI: 0.65–0.70 1-year OPERA score: AUC 0.74, 95%CI: 0.73–0.75 NHFS: AUC 0.70, 95%CI: 0.69–0.71	30-day OPERA score: The plots showed overestimating with the moderate to high-risk patients NHFS: The plots showed overestimating with the moderate to high-risk patients 1-year: Not reported
Doherty 2021 [25]	NHFS	In-hospital, 30-day, and 120-day mortality	In-hospital: C-statistic = 0.684, 95%CI: 0.650–0.718 30-day: C-statistic = 0.690, 95%CI: 0.652–0.727 120-day: C-statistic = 0.676, 95%CI: 0.641–0.710	The plots showed overestimating with the higher-risk patients
Etscheidt 2021 [26]	MPS	In-hospital, 30-day, 60-day and 90-day mortality	In-hospital: AUC 0.80, 95%CI: 0.74–0.86 30-day: AUC 0.73, 95%CI: 0.69–0.78 60-day: AUC 0.74, 95%CI: 0.70–0.78 90-day: AUC 0.74, 95%CI: 0.70–0.77	30-day: The curve showed good calibration Not reported other time points
Jonsson 2018 [31]	POSSUM P-POSSUM NHFS	30-day mortality	POSSUM: AUC 0.66, 95%CI: 0.59–0.72 P-POSSUM: AUC 0.66, 95%CI: 0.59–0.72 NHFS: AUC 0.67, 95%CI: 0.59–0.74	POSSUM: O:E ratio deaths = 0.90 (no CI reported) P-POSSUM: O:E ratio deaths = 0.98, 95%CI: 0.76–1.21 NHFS: O:E ratio deaths = 0.79, 95%CI: 0.58–1.00
Karres 2022 [32]	NHFS HEMA	30-day, 1-year, and 5-year mortality	30-day NHFS: AUC 0.77, 95%CI: 0.67–0.86 HEMA: AUC 0.73, 95%CI: 0.61–0.85 1-year NHFS: AUC 0.74, 95%CI: 0.66–0.81 HEMA: AUC 0.78, 95%CI: 0.71–0.85 5–year NHFS: AUC 0.78, 95%CI: 0.73–0.84 HEMA: AUC 0.80, 95%CI: 0.74–0.85	30-day NHFS: Intercept = 0.00 and slope = 1.23 HEMA: Intercept = − 1.62 and slope = 0.39, representing both systematic overestimation and severe overfitting. 1 and 5 years showed no significant lack of fit in the Hosmer-Lemeshow goodness-of-fit test
Marufu 2016 [33]	NHFS	30-day mortality	AUC 0.71, 95%CI: 0.67–0.75	The plots showed overestimating with the higher-risk patients
Olsen 2021 [35]	NHFS	30-day mortality	AUC 0.64, 95%CI: 0.61–0.68	The plots showed underestimating in most ranges
Oosterhoff 2023 [37]	SORG femoral neck fracture mortality algorithm	90-day and 2-year mortality	90-day C-statistic = 0.67, 95%CI: 0.62–0.71 2-year C-statistic = 0.67, 95%CI: 0.65–0.70	90-day: Intercept = 0.18, 95%CI: 0.02–0.35 and slope = 0.92, 95%CI: 0.67–1.17 2-year: Intercept = 0.50, 95%CI: 0.40–0.61 and slope = 0.90, 95%CI: 0.74–1.04
Rushton 2015 [38]	NHFS	In-hospital mortality	Not reported	The ratio of observed and predicted probability showed an overestimation with the low risk patients
Schuijt 2022 [41]	U-HIP	In-hospital mortality	C-statistic = 0.74, 95%CI: 0.72–0.76	Intercept = 0.015 and slope = 0.780
Tilkeridis 2018 [42]	NHFS Modified NHFS	30-day mortality	NHFS: AUC 0.83 Modified NHFS: AUC 0.84	NHFS: The plots showed good calibration. Modified NHFS: The plots showed good calibration
van Zeeland 2011 [44]	POSSUM	In-hospital mortality	AUC 0.83, 95%CI: 0.76–0.89	The plots showed good calibration

*AHFS*^*90*^ Almelo Hip Fracture Score 90, *BHFS* Brabant hip fracture score, *GeRi-Score* Geriatrics at Risk Score, *HEMA* Hip Fracture Estimator of Mortality Amsterdam, *MPS* mortality prediction score, *NHFS* Nottingham Hip Fracture Score, *POSSUM* Physiological and Operative Severity Score for the enUmeration of Mortality and Morbidity, *P-POSSUM* Portsmouth-Physiological and Operative Severity Score for the enUmeration of Mortality and Morbidity, *U-HIP* Utrecht Hip algorithm

### Risk of bias assessment and applicability

Table 3 summarizes the results of our analyses of the risk of bias and applicability for all prognostic models. The assessment considered 33 analyses involving the development and validation of prognostic models. According to PROBAST, all of the development studies were observed to have a high risk of bias. Elven prediction models [23, 24, 27–30, 36, 39, 40, 43, 45] (92%) exhibited a high risk of bias in the participant domain, primarily because most of the models used existing data from databases that were not collected specifically to develop prognostic models. Nine prognostic models [23, 24, 27, 29, 30, 34, 39, 40, 45] (75%) were judged to have a high risk of bias in the analysis domain, largely due to a small sample size, failure to included all participants in the analysis, and/or inappropriate handling of missing data.

**Table 3:** Risk of bias assessment adapted prediction model risk of bias assessment tool

Study (ref.)		Risk of bias				Applicability			Overall	
		Participants	Predictors	Outcome	Analysis	Participants	Predictors	Outcome	Risk of bias	Applicability
Development										
Cary Jr 2021 [23]	Cary Jr. clinical prediction model	High	Low	Low	High	Low	Low	Low	High	Low
Cenzer 2016 [24]	Cenzer clinical prediction model	High	High	Low	High	Low	Low	Low	High	Low
Fu, 2021 [27]	Fu clinical prediction model	High	High	Low	High	Low	High	Low	High	High
Goubar 2023 [28]	Stratify-Hip Algorithm	High	Low	Low	Low	Low	Low	Low	High	Low
Hjelholt 2022 [29]	Hjelholt clinical prediction model	High	High	Low	High	Low	Low	Low	High	Low
Jiang 2005 [30]	Jiang Risk score	High	High	Low	High	Low	Low	Low	High	Low
Nijmeijer 2023 [34]	AHFS^90^	Low	Low	Low	High	Low	Low	Low	High	Low
Oosterhoff 2022 [36]	SORG femoral neck fracture mortality algorithm	High	High	Low	Low	Low	Low	Low	High	Low
Sanz-Reig 2024 [39]	RNFC model	High	Low	Low	High	Low	Low	Low	High	Low
Schoeneberg 2023 [40]	GeRi-Score	High	Low	Low	High	Low	Low	Low	High	Low
Yoo 2022 [43]	Yoo clinical prediction model	High	Low	Low	Low	Low	High	Low	High	High
van de Ree 2019 [45]	BHFS	High	High	Low	High	Low	Low	Low	High	Low
Validation										
Arjan 2023 [22]	NHFS	Low	Low	Low	Low	Low	Low	Low	Low	Low
Doherty 2021 [25]		High	Low	Low	Low	Low	Low	Low	High	Low
Jonsson 2018 [31]		Low	Low	Low	High	Low	Low	Low	High	Low
Karres 2022 [32]		Low	Low	Low	Low	Low	Low	Low	Low	Low
Marufu 2016 [33]		Unclear	Low	Low	High	Low	Low	Low	High	Low
Olsen 2021 [35]		High	Low	Low	Low	Low	Low	Low	High	Low
Rushton 2015 [38]		High	Low	Low	High	Low	Low	Low	High	Low
Tilkeridis 2018 [42]		High	Low	Low	High	Low	Low	Low	High	Low
Jonsson 2018 [31]	POSSUM	Low	Low	Low	High	Low	Low	Low	High	Low
van Zeeland 2011 [44]		High	Low	Low	High	Low	Low	Low	High	Low
Arjan 2023 [22]	OPERA score	Low	Low	Low	Low	Low	Low	Low	Low	Low
Etscheidt 2021 [26]	MPS	Low	Low	Low	High	Low	Low	Low	High	Low
Goubar 2023 [28]	Stratify-Hip Algorithm	High	Low	Low	Low	Low	Low	Low	High	Low
Jiang 2005 [30]	Jiang Risk score	High	High	Low	High	Low	Low	Low	High	Low
Jonsson 2018 [31]	P-POSSUM	Low	Low	Low	High	Low	Low	Low	High	Low
Karres 2022 [32]	HEMA	Low	Low	Low	Low	Low	Low	Low	Low	Low
Oosterhoff 2023 [37]	SORG femoral neck fracture mortality algorithm	High	High	Low	Low	Low	Low	Low	High	Low
Sanz-Reig 2024 [39]	RNFC model	High	Low	Low	High	Low	Low	Low	High	Low
Schoeneberg 2023 [40]	GeRi-Score	High	Low	Low	High	Low	Low	Low	High	Low
Schuijt 2022 [41]	U-HIP	High	Low	Low	Low	Low	Low	Low	High	Low
Tilkeridis 2018 [42]	Modified NHFS	High	Low	Low	High	Low	Low	Low	High	Low

*AHFS*^*90*^ Almelo Hip Fracture Score 90, *BHFS* Brabant hip fracture score, *GeRi-Score* Geriatrics at Risk Score, *HEMA* Hip Fracture Estimator of Mortality Amsterdam, *MPS* mortality prediction score, *NHFS* Nottingham Hip Fracture Score, *POSSUM* Physiological and Operative Severity Score for the enUmeration of Mortality and Morbidity, *P-POSSUM* Portsmouth-Physiological and Operative Severity Score for the enUmeration of Mortality and Morbidity, *U-HIP* Utrecht Hip algorithm

Four (19%) prognostic models [22, 33] from three validation studies were shown to have a low risk of bias, particularly in the predictor and outcome domains, where most models were assessed to have a low risk of bias. Twelve (57%) prognostic models [25, 30, 35, 37–42, 44] from 11 studies had a high risk of bias in the participant domain because they were secondary analyses of data from other studies. Twelve (57%) prognostic models [26, 30, 31, 33, 38–40, 42, 44] from eight studies had a high risk of bias in the analysis domain, mainly due to small sample sizes and issues with model performance evaluation.

Two studies (17%) [27, 43] demonstrated high applicability concerns in development studies, while all of the studies were considered to involve low concern in terms of overall applicability to the review questions (Table 3). Regarding predictors, two studies (17%) [27, 43] were assessed to have a high applicability concern. These concerns related inconsistencies with the review question in terms of predictor definition, delayed timing of the predictor measurement, and a large standard deviation in the measurement timing relative to the review question.

### Synthesis results

**Fig. 2:**
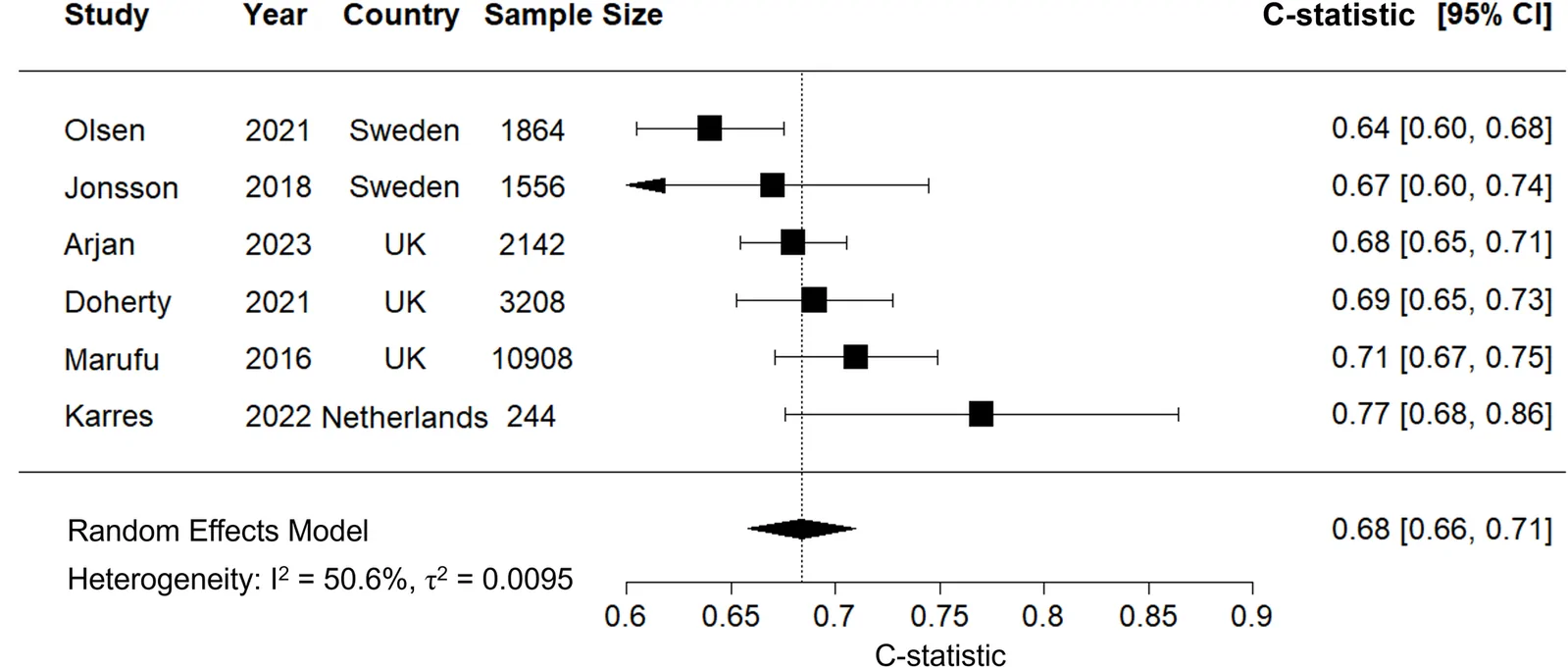
Forest plots of the C-statistics in the studies in which the Nottingham Hip Fracture Score (NHFS) and 30-day mortality were examined. Caption: C-statistics were logit-transformed prior to meta-analysis and back-transformed to the original scale for display

Seven studies [25, 31–33, 35, 38, 42] had external validation of 30-day mortality with the NHFS. We performed a meta-analysis of six of these studies, excluding the seventh due to its inadequate discrimination measure, as no confidence interval was reported and the data could not be obtained from the corresponding author. A random-effects model was used for the meta-analysis, and the results showed that the pooled C-statistic was 0.68 (95%CI: 0.66–0.71) (Fig. 2). Moderate heterogeneity was observed across studies (I² = 50.6%). There was little variation among studies, and the discrimination accuracy was similar to that of the development studies. Sensitivity analysis including studies with supplemented data yielded a pooled C-statistic of 0.71 (95% CI: 0.66–0.76), which was consistent with the primary analysis.

We performed a narrative synthesis for other time points because a meta-analysis was impossible. Multiple validation studies focused on in-hospital and 1-year mortality. For in-hospital mortality, we submitted eight studies (seven models) [25, 26, 28, 30, 38, 40, 41, 44] to our five-step process, and the results revealed that none of the studies showed high concerns about applicability, two of the eight studies [30, 38] showed no or incomplete reporting of performance measures, and one of six studies [25] showed poor calibration. Among the five studies that demonstrated possibly helpful discrimination, none showed a risk of bias with a major impact. This left two studies (2/5), using the POSSUM [44] and a mortality prediction score (MPS) [26], with clearly helpful discrimination measures (Supplemental Table S2).

For 1-year mortality, we submitted the three studies (four models) [22, 30, 32] to our five-step process. None of the three showed high concerns about applicability, one of the three studies [31] provided no report of performance measures, and one of two studies [22] showed poor calibration. The remaining NHFS study [32] demonstrated possibly helpful discrimination and no major risk of bias concerns (Supplemental Table S3).

## Discussion

We conducted a systematic literature search that identified 24 studies, eight development models, and 13 validation models tested for predicting mortality among older adults with hip fractures [22–45]. Most of the model development studies had high accuracy but underwent only internal validation and were at high risk of bias. The validation studies were often conducted as a geographical validation of commonly used models, i.e., the NHFS [6] and POSSUM [7]. The performance of these models showed good calibration and moderate discrimination; only a few models had a low risk of bias. Our synthesis showed that the NHFS for predicting 30-day and 1-year mortality and the POSSUM and MPS for predicting in-hospital mortality appear promising for use in clinical settings.

In this systematic review, the most commonly used time point was 30-day post-surgical mortality, and a meta-analysis could be performed only for the NHFS. Our finding that the NHFS was the most widely used model for predicting 30-day mortality is consistent with previous research [3, 5]. Although a meta-analysis could be performed, the pooled C-statistic was 0.68 (95%CI: 0.66–0.71), indicating poor to modest discriminative performance and falling short of a level that would be considered clinically useful, and its accuracy was inferior to that of the development study by Maxwell et al. [6] The NHFS is a simple prognostic model that assigns scores based on seven variables that can be measured at a patient’s admission [6]. Therefore, the NHFS helps clinicians stratify risk, guide perioperative management, and support communication with patients and families. Also, since NHFS has been in development for some time, its performance should be considered in relation to the current healthcare system. While some updates have been performed since the development, the original NHFS was also externally validated in several countries including Sweden [35], the Netherlands [46], and Greece [42]. Currently, the NHFS is recommended for predicting 30-day mortality, but its discriminatory ability remains limited. In future research, it is desirable to externally validate the high-performance prognostic models developed in recent years [23, 28, 34, 39, 45]. If a new model is developed, it should have a clear purpose and be carefully designed to minimize the risk of bias.

Other time points for external validation were poorly described and could not be meta-analyzed. Despite the limited evidence, clinical decision-making occurs on a daily basis, and we therefore synthesized the results based on the criteria described by Naye et al. [21] and provided recommendations. For in-hospital mortality, seven prognostic models [25, 26, 28, 30, 38, 40, 41, 44] were externally validated at least once. Among these models, POSSUM and MPS demonstrated acceptable calibration and high discrimination accuracy. Although the studies underpinning these models were rated as having a high risk of bias according to PROBAST, we considered the likely impact of the identified bias domains on model performance when evaluating their clinical usefulness. Both POSSUM and MPS are used to predict mortality, but neither is specific to hip fractures. While POSSUM is designed for surgical patients and can predict postoperative mortality and morbidity, it is relatively complex because it requires numerous physiological and operative variables. The more recently developed models [28, 40, 43] for predicting in-hospital mortality in patients with hip fractures had a high risk of bias, and unfortunately, their discriminatory performance was not significantly different from that of POSSUM and MPS. Therefore, further external validation of these two models is recommended, as there are currently no models that predict in-hospital mortality specifically for patients with hip fractures.

We observed that the studies’ long-term time points for estimating mortality ranged from 6 months to 5 years, with 1 year the most frequent time point. Prognostic models were developed at the 1-year time point, but only four models [22, 30, 32] were externally validated. The NHFS was the preferred model for clinical use because of its acceptable calibration, high discrimination, and low risk of bias. This point was also not clear in the existing systematic review [3–5]. However, since one year is a relatively long time, older hip fracture patients with multimorbidity and multiple comorbidities may have died from other causes. In addition to mortality, the core outcome for individuals with a hip fracture include mobility, activities of daily living, health-related quality of life, and pain [2]. Physicians and clinicians need to be aware of these additional core outcomes when patients’ long-term survival is considered. The development and validation of prognostic models for these outcomes are far less advanced than those for mortality [47] and remains a challenge.

Data from national databases and registry studies formed the basis of many studies included in this systematic review. Some studies included > 10,000 participants. Research using large-scale data is expected to increase in the future, but it is important to note that many studies do not have the primary aim of developing prognostic models. We also observed that many studies had a high risk of bias in the analysis domain. Efforts are being made to improve the quality of prognostic model research, including the development of bias risk assessment tools (e.g., PROBAST) [18], and in recent years, the reporting guidelines have been updated from TRIPOD to TRIPOD + AI [48]. It is desirable to make effective use of these tools to develop prognostic models that support clinical decision-making while minimizing the risk of bias. When considering the application of prognostic models to clinical practice, it is necessary to conduct an impact assessment after the models’ verification and to conduct further implementation research. However, none of the studies we identified performed impact assessments on mortality outcomes in older adults with hip fractures, and further investigations are thus necessary.

We should consider new candidate predictors for future prognostic model development. Recently, it has become clear that operative time and surgeon experience are associated with risk factors for mortality and complications in patients with hip fractures [49]. These factors are important because they may reflect medical advances resulting from changes in surgical instruments and procedures. In addition, patient-related risk factors such as age have been shown to substantially affect complication rates and functional outcomes [50]. Although age is included in many prognostic models, further stratification may be necessary in the current aging population. Integrating such findings will hopefully provide additional clinical context and help position prognostic models within the broader framework of risk stratification and outcome prediction.

A strength of the present systematic review is that it used a meta-analysis or narrative synthesis to identify the predictive models recommended for application at each time point. In addition, we rigorously evaluated the risk of bias in the prognostic model using PROBAST as a benchmark.

Several limitations of our systematic review should be noted. We included only studies in the validation phase (internal or external). This criterion was defined according to the recommendations of the TRIPOD statements but may have excluded studies that were only highly precise in the development phase. Another study limitation is that we were unable to assess the quality of reporting based on the updated TRIPOD + AI statement. Moreover, although this systematic review provides recommendations based on prognostic models that had been externally validated at least once, most of the included studies were at high risk of bias. This substantially undermines the reliability and generalizability of these models and limits their applicability in routine clinical practice.

In conclusion, our systematic review identified 21 different prognostic models that met the inclusion criteria: eight models were in the development stage [23, 24, 27, 29, 34, 36, 43, 45] and 13 were in the validation stage [22, 25, 26, 28, 30–33, 35, 37–42, 44]. The prognostic models were designed to include predictors that are available before and after surgery in clinical settings and patient populations in various countries. The most thoroughly investigated prognostic model was the NHFS. The prognostic models identified in this systematic review showed acceptable calibration and moderate discrimination but did not adequately minimize biases due to methodological weaknesses and were judged high-risk by PROBAST. Based on the current evidence and synthesis results, we recommend the NHFS for predicting 30-day and 1-year mortality and the POSSUM and MPS for predicting in-hospital mortality among older adults with a hip fracture; however, given the overall high risk of bias, these models should be used with caution and only as adjunctive tools rather than for independent clinical decision-making. Future prognostic models should focus on external validation, be conducted and reported in accord with the TRIPOD + AI statement, and incorporate the considerations outlined here in order to minimize the risk of bias.

## Supplementary Information

Supplementary Material 1.

## Data Availability

The datasets used and/or analyzed during the current study are available from the corresponding author upon reasonable request.

## Abbreviations

AUC: area under the receiver operating characteristic curve
CHARMS: Checklist for Critical Appraisal and Data Extraction for Systematic Reviews of Prediction Modelling Studies
CI: Confidence intervals
MPS: mortality prediction score
NHFS: Nottingham Hip Fracture Score
POSSUM: Physiological and Operative Severity Score for the enUmeration of Mortality and morbidity
PROBAST: Prediction model Risk of Bias Assessment Tool
PRISMA: Preferred Reporting Items for Systematic Review and Meta-Analyses
TRIPOD-SRMA: Transparent Reporting Of multivariable prediction models for Individual Prognosis Or Diagnosis checklist for Systematic Reviews and Meta-Analyses
