# Supplementary material for: Models for predicting short- to long-term mortality in older adults with hip fractures in clinical practice: a systematic review and meta-analysis
Source: BMC Geriatr. 2026 Jun 23;26:1142. doi: 10.1186/s12877-026-07814-y (PMC13548648; doi:10.1186/s12877-026-07814-y)
Supplement: Supplementary file 1 [file 12877_2026_7814_MOESM1_ESM.docx]

**Supplemental Table S1: Full search strings and number of results**

**PubMed**

| #1 | (“Hip Fractures”[Mesh] OR “Trochanteric Fracture” OR “Intertrochanteric Fracture” OR “Subtrochanteric Fracture” OR “Femoral Fractures”[Mesh] OR “Femoral Neck Fractures”[Mesh] OR “Femoral Neck Fracture” OR “Femur Neck Fractures” OR “Femur Neck Fracture”) | 48,525 |
| --- | --- | --- |
| #2 | ((((Validat*[tw] OR Predict*[ti] OR Rule*[tw]) OR (Predict*[tw] AND (Outcome*[tw] OR Risk*[tw] OR Model*[tw])) OR ((History[tw] OR Variable*[tw] OR Criteria[tw] OR Scor*[tw] OR Characteristic*[tw] OR Finding*[tw] OR Factor*[tw]) AND (Predict*[tw] OR Model*[tw] OR Decision*[tw] OR Identif*[tw] OR Prognos*[tw])) OR (Decision*[tw] AND (Model*[tw] OR Clinical*[tw] OR "Logistic Models"[mh])) OR (Prognostic[tw] AND (History[tw] OR Variable*[tw] OR Criteria[tw] OR Scor*[tw] OR Characteristic*[tw] OR Finding*[tw] OR Factor*[tw] OR Model*[tw])))) OR (((((((((((("ROC Curve"[Mesh]) OR stratificat*[tw]) OR discriminat*[tw]) OR c statistic*[tw]) OR Area under the curve*[tw]) OR AUC[tw]) OR Calibrat*[tw]) OR Indices[tw]) OR Algorithm*[tw]) OR Multivariable*[tw])) OR ((Predict*[tw] OR Predictive value of tests[mh] OR Scor*[tw] OR Observ*[tw] OR Observer variation[mh])))) | 11,236,340 |
| #3 | (“Mortality”[Mesh] OR “Death Rate” OR “Death Rates” OR “Death”[Mesh]) | 597,252 |
| #4 | #1 AND #2 AND #3 | 1,071 |
| #5 | #4 NOT Randomized controlled trial[pt] NOT Review[pt] | 1,002 |
| #6 | #5 AND "last 20 years"[PDat] | 852 |

**CINAHL**

| #1 | (MH"Hip Fractures" OR MH"Trochanteric Fracture" OR MH"Intertrochanteric Fracture" OR MH"Subtrochanteric Fracture" OR MH"Femoral Fractures" OR MH"Femoral Neck Fractures" OR MH"Femoral Neck Fracture" OR MH"Femur Neck Fractures" OR MH"Femur Neck Fracture") | 18,130 |
| --- | --- | --- |
| #2 | ((((Validat* OR Predict* OR Rule*) OR (Predict* AND (Outcome* OR Risk* OR Model*)) OR ((History OR Variable* OR Criteria OR Scor* OR Characteristic* OR Finding* OR Factor*) AND (Predict* OR Model* OR Decision* OR Identif* OR Prognos*)) OR (Decision* AND (Model* OR Clinical* OR MH"Logistic Models")) OR (Prognostic AND (History OR Variable* OR Criteria OR Scor* OR Characteristic* OR Finding* OR Factor* OR Model*)))) OR ((((((((((((MH"ROC Curve") OR stratificat*) OR discriminat*) OR c statistic*) OR Area under the curve*) OR AUC) OR Calibrat*) OR Indices) OR Algorithm*) OR Multivariable*)) OR ((Predict* OR Predictive value of tests OR Scor* OR Observ* OR MH"Observer variation")))) | 2,504,646 |
| #3 | (MH"Mortality" OR MH"Death Rate" OR MH"Death Rates" OR MH"Death") | 54,006 |
| #4 | #1 AND #2 AND #3 | 309 |
| #5 | #4 NOT “Randomized controlled trial” NOT Review | 306 |
| #6 | Filtered the time period designation to 20 years | 291 |

**Cochrane Library**

| #1 | (mh"Hip Fractures" OR "Trochanteric Fracture" OR "Intertrochanteric Fracture" OR "Subtrochanteric Fracture" OR mh"Femoral Fractures" OR mh"Femoral Neck Fractures" OR "Femoral Neck Fracture" OR "Femur Neck Fractures" OR "Femur Neck Fracture") | 26,784 |
| --- | --- | --- |
| #2 | (((((tw"Validate" OR ti"Predicte" OR tw"Rule") OR (tw"Predicte" AND (tw"Outcome" OR tw"Risk" OR tw"Model"))) OR ((tw"History" OR tw"Variable" OR tw"Criteria" OR tw"Score" OR tw"Characteristic" OR tw"Finding" OR tw"Factor") AND (tw"Predicte" OR tw"Modele" OR tw"Decision" OR tw"Identife" OR tw"Prognos"))) OR (tw"Decision" AND (tw"Model" OR tw"Clinical" OR mh"Logistic Models"))) OR (tw"Prognostic" AND (tw"History" OR tw"Variable" OR tw"Criteria" OR tw"Score" OR tw"Characteristic" OR tw"Finding" OR tw"Factor" OR tw"Model"))) OR (tw"Predict" OR mh"Predictive value of tests" OR tw"Score" OR tw"Observe" OR mh"Observer variation") | 367,089 |
| #3 | (Mortality OR Death Rate OR Death Rates OR Death) | 177,122 |
| #4 | #1 AND #2 AND #3 | 4,883 |
| #5 | #4 NOT (pt"Randomized controlled trial" OR pt"Review") | 2 |

**Japanese database**

**Ichushi (医学中央雑誌)**

| #1 | ((股関節部骨折/TH or 股関節骨折/AL) or 大腿骨近位部骨折/AL or (大腿骨頸部骨折/TH or 大腿骨頸部骨折/AL) or (大腿骨頸部骨折/TH or 大腿骨頚部骨折/AL) or (股関節部骨折/TH or 大腿骨転子部骨折/AL)) | 38,528 |
| --- | --- | --- |
| #2 | (予測モデル/AL or 臨床予測モデル/AL or (臨床意思決定ルール/TH or 臨床意思決定ルール/AL)) | 2,003 |
| #3 | (予測モデル/AL or 臨床予測モデル/AL or (臨床意思決定ルール/TH or 臨床意思決定ルール/AL)) | 262,229 |
| #4 | #1 AND #2 AND #3 | 7 |

**CiNii**

| #1 | (股関節骨折 OR 大腿骨近位部骨折 OR 大腿骨頸部骨折 OR 大腿骨頚部骨折 OR 大腿骨転子部骨折) | 9,609 |
| --- | --- | --- |
| #2 | (予測モデル OR 臨床予測モデル OR 臨床意思決定ルール) | 8,826 |
| #3 | (死亡率 OR 死亡) | 62,683 |
| #4 | #1 AND #2 AND #3 | 0 |

**Supplemental Table S2. Results of narrative synthesis in hospital mortality**

|  |  | **Five-step process to identify a prognostic model with clinical value** | | | | |
| --- | --- | --- | --- | --- | --- | --- |
| **Study** | **Prediction model** | **1**  **Applicability** | **2**  **Performance measures** | **3**  **Calibration** | **4**  **Discrimination** | **5**  **Risk of bias** |
| Schuijt, 2022 | U-HIP | Y | Y | PY | AUC = 0.74 | Y |
| Jiang, 2005 | Jiang clinical prediction model | Y | N |  |  |  |
| Doherty, 2021 | NHFS | Y | Y | PN |  |  |
| Etscheidt, 2020 | MPS | Y | Y | Y | **AUC = 0.80** | Y |
| Schoeneberg, 2023 | GeRi-Score | Y | Y | PY | AUC = 0.72 | PY |
| van Zeeland, 2011 | POSSUM | Y | Y | PY | **AUC = 0.83** | PY |
| Rushton, 2015 | NHFS | Y | N |  |  |  |
| Goubar, 2023 | Stratify-Hip Algorithm | Y | Y | Y | AUC = 0.73 | Y |

AUC, area under the curve; GeRi-Score, Geriatrics at Risk Score; MPS, mortality prediction score; N, no; NHFS, Nottingham hip fracture score; PN, probably no; PY, probably yes; POSSUM, Physiological and Operative Severity Score for the enUmeration of Mortality and Morbidity; Y, yes; U-HIP, Utrecht Hip algorithm.

**Supplemental Table S3. Results of narrative synthesis in 1-year mortality**

|  |  | **Five-step process to identify a prognostic model with clinical value** | | | | |
| --- | --- | --- | --- | --- | --- | --- |
| **Study** | **Prediction model** | **1**  **Applicability** | **2**  **Performance measures** | **3**  **Calibration** | **4**  **Discrimination** | **5**  **Risk of bias** |
| Jiang, 2005 | Jiang clinical prediction model | Y | N |  |  |  |
| Karres, 2022 | NHFS  HEMA | Y | Y | NHFS: PY  HEMA: PN | **AUC = 0.77** | Y |
| Arjan, 2023 | OPERA  NHFS | Y | Y |  |  |  |

AUC, area under the curve; HEMA, Hip fracture Estimator of Mortality Amsterdam, N, no; NHFS, Nottingham hip fracture score; OPERA, Older Persons' Emergency Risk Assessment; PN, probably no; PY, probably yes; Y, yes.
